# Antibiotic impregnated cement coated intramedullary nail (ACCIN) using bronchoscopy tubing: technical tips, case series and a review of the literature

**DOI:** 10.1007/s00590-023-03668-x

**Published:** 2023-08-28

**Authors:** Christy Graff, Tanishq Mathur

**Affiliations:** 1 The University of Adelaide, Adelaide, SA Australia; 2 Department of Orthopaedics, Royal Adelaide Hospital, Adelaide, SA Australia; 3 Department of Orthopaedics, Women’s and Children’s Hospital, Adelaide, SA Australia

**Keywords:** Antibiotic impregnated cement coated intramedullary nail, ACCIN, Infected non-union, Tibial osteomyelitis

## Abstract

Antibiotic impregnated cement coated intramedullary nails (ACCINs) have been used in clinical practice for many years and have been shown to help eradicate infection in tibial osteomyelitis while providing stability. We present a novel technique for preparation using bronchoscopy tubing, as well as technical tips and a review of the literature, for ease of preparation and potential subsequent retrieval.

## Introduction

The use of antibiotic cement coated interlocking nails (ACCINs) for infected delayed or non-unions of tibial shaft fractures has been established as a safe and practical method of eradicating infection while simultaneously providing stability [1–4]. ACCIN can be performed simultaneously with other management of this challenging condition, such as surgical debridement, adequate soft tissue coverage, systemic administration of specific antimicrobials and/or dead space management [5]. Previous methods of treatment such as first removing infected hardware, eradicating infection, followed by definitive fixation months later once infection has been cleared, present challenges such as prolonged periods of non-weight bearing which can be severely functionally limiting for the patient.

ACCIN allows removal of infected hardware and local administration of antibiotics directly to the source of infection. Higher doses of antibiotics can be administered without systemic toxicity with this approach [6], with a higher minimum inhibitory concentration to treat biofilm than using systemic antibiotics alone [7]. It is also useful in patients who are non-compliant with post-operative antibiotic treatment as well as patients who are not ideal candidates for external fixators or those who do not want an external fixator [8]. One challenge in this technique remains the lack of consensus on the optimal preparation method of these nails [2]. Removal can be difficult due to the risk of delamination of cement; a successful ACCIN removal usually starts with the preparation [9].

We propose a novel method of preparing ACCIN using bronchoscopy tubing. 8 mm intramedullary nails were coated with COPAL G + C and/or COPAL G + V cement with the Heraeus Palamix uno system with the aid of sterile, individually packed Ambu bronchoscopy tubing. The practical considerations of using bronchoscopy tubing, pre-operative work-up for this operation, stepwise technique and 3 clinical cases are highlighted below.

## Methods

### Practical considerations

**Fig. 1:**
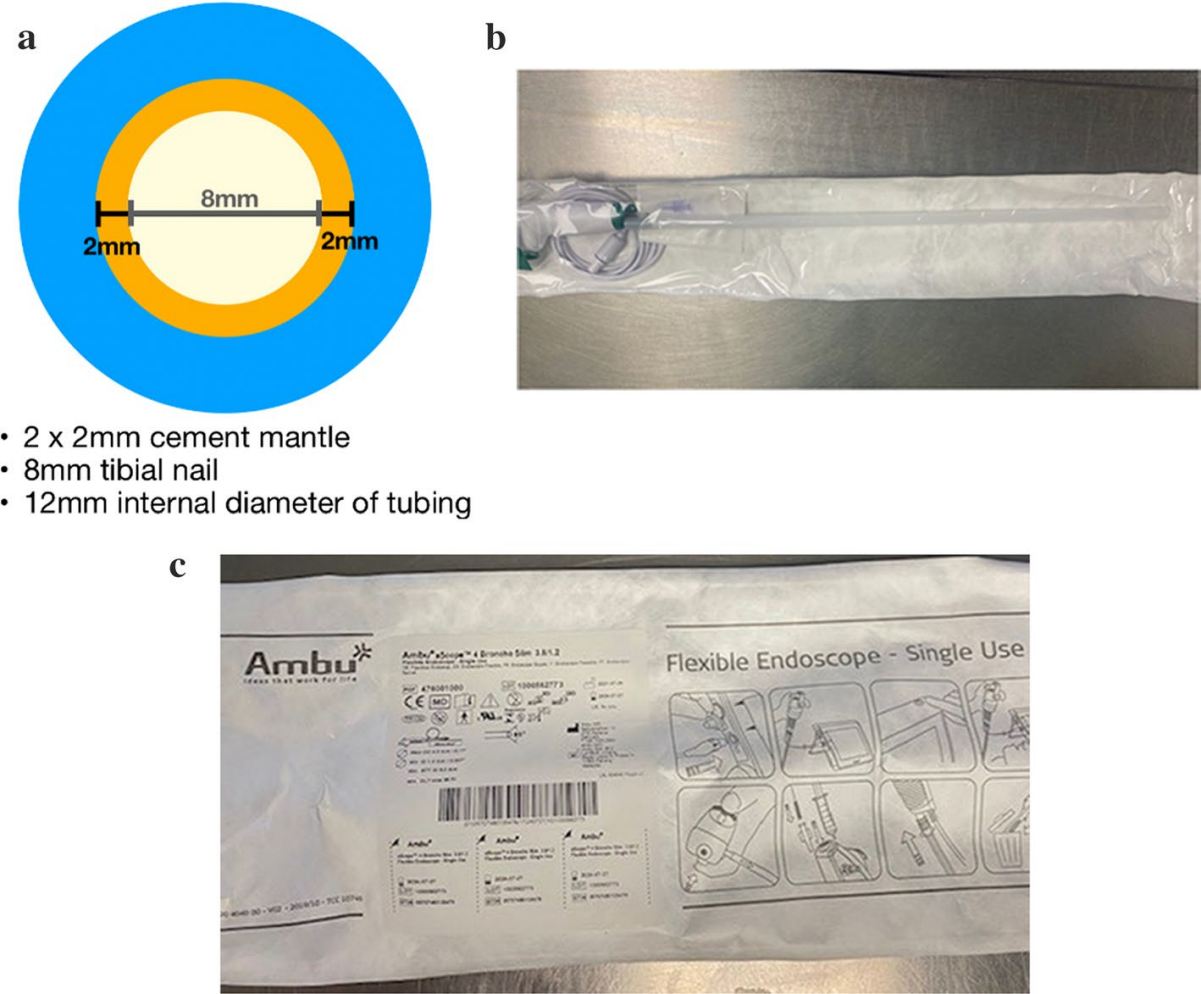
Bronchoscopy tubing. **a** Dimensions of the bronchoscopy tubing in relation to the cement nail. **b** Individually wrapped and sterile bronchoscopy pack. **c** Commercial brand of bronchoscopy tubing

Several materials were considered when developing a method of preparing ACCIN at our institution; the bronchoscopy tubing was the most appropriate. The internal diameter of the bronchoscopy tubing was the most important factor in our choice. To construct an ACCIN, a 2 mm circumferential cement mantle either side of the smallest diameter tibial nail (8 mm) was required; hence, the 12 mm internal diameter of the bronchoscopy tubing was optimal (see Fig. 1a Dimensions of the bronchoscopy tubing in relation to the cement nail).

The ideal mould for these nails required rigidity to maintain the shape of the nail but material that could be split easily to be separated from the nail. The bronchoscopy tubing also came in a sterilised package. This, combined with the use of proper sterile technique when constructing these nails using the bronchoscopy tubing, maximised the sterility of the nail and the procedure itself (see Fig. 1b Individually wrapped and sterile bronchoscopy pack). The Ambu bronchoscopy tubing comes in a sterilised package (see Fig. 1c Commercial brand of bronchoscopy tubing), and the commercial cost of this set is $AUD289.

There is literature on other materials being used for construction of ACCIN. These materials include chest drains, dilatation and curettage tubing, specialised metal moulding and commercially available silicon tubing. Each of these materials were considered but presented specific challenges. The chest drains were available to an internal diameter of 9.6 mm which was too small for a 2 mm cement mantle around an 8 mm nail, while the dilatation and curettage tubing was too large with an internal diameter of 14 mm. This may be suitable for a femoral ACCIN. Metal moulding was difficult to obtain as well as expensive. Silicon tubing commercially available at hardware stores was not approved for sterilisation from CSSD at our institution.

### Pre-operative considerations

The pre-operative preparation included a CT evaluating the need for sequestrectomy, assessing the delayed or non-union and/or bone loss, and evaluating the intramedullary canal diameter to guide the choice of nail; an 8 mm tibial nail was used for all three of our clinical cases. A computerised tomography angiogram (CTA) was performed if there was any question of vascular disease or if a flap was being considered.

All patients also had an infectious diseases review regarding targeted antibiotics and dosing for cement. Targeted antibiotics were a combination of gentamicin + clindamycin, gentamicin + vancomycin or both. These were combined with high viscosity, radio-opaque revision bone cement (COPAL G + C and COPAL G + V bone cements). Other consults included vascular or plastic surgery if required.

### Operative technique

1. The infected hardware is removed through previous incisions.
2. Dead bone and sequestra are debrided to healthy bleeding bone.
3. The reamer–irrigator–aspirator (RIA Depuy Synthes) is used to irrigate the canal and ream the tibia 14 mm to prevent cement delamination from the tibia when placing the nail.
  - 14 mm is the narrowest diameter to be reamed to; if this cannot be achieved, a different technique needs to be utilised.
4. A new prep and drape, and new instruments are used for preparation of ACCIN.

### Preparation of the ACCIN

**Fig. 2:**
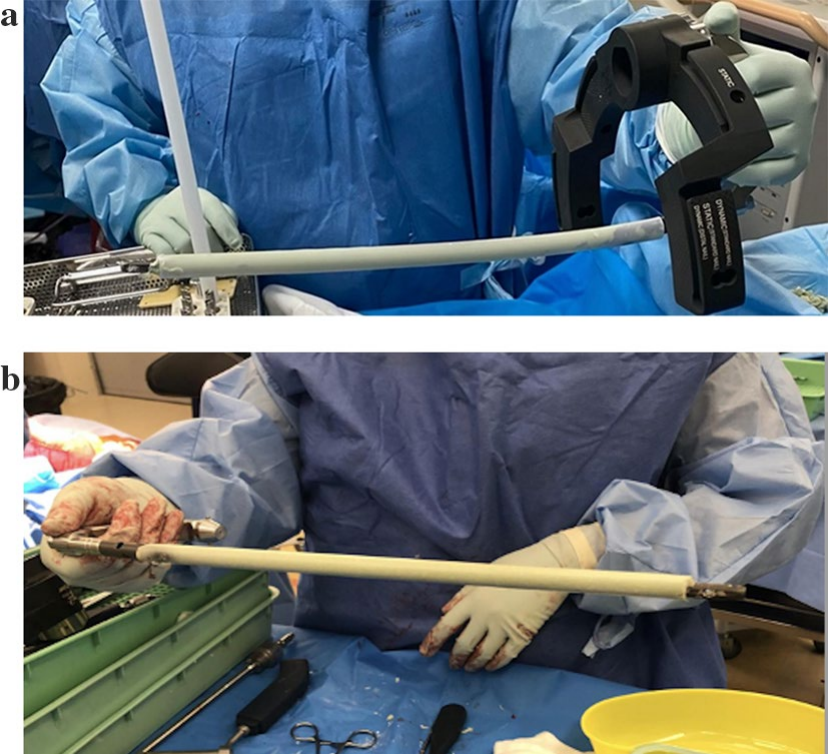
Intraoperative preparation of ACCIN using bronchoscopy tubing. **a** Prepared ACCIN with tubing over the cement. **b** Final ACCIN attached to jig ready for insertion

1. The bronchoscopy tubing is cut to a predetermined length to leave the distal and proximal locking screw holes of the nail outside the tubing.
2. Antibiotic powder is hand mixed with 2 × Palacos COPAL G + C or COPAL G + V bone cement using Palamix uno system.
3. The prepared cement is inserted to fill the entire cut length of the bronchoscopy tubing using a Palamix uno cement gun.
  - It is important that the cement gun nozzle is long enough to fill the whole tube; otherwise, part of the nail will not be coated and the coating will not be uniform.
4. The tibial nail is inserted into the tubing while the cement is pliable.
5. The proximal and distal locking holes are left out of the tubing. Any stray cement is cleared from the proximal and distal locking holes (see Fig. 2a Prepared ACCIN with tubing over the cement).
6. Once the cement is set, the tubing is cut longitudinally with a 20 blade scalpel and slides easily off the ACCIN.
7. Once the tubing is removed from the ACCIN, the nail is ready to insert (see Fig. 2b Final ACCIN attached to jig ready for insertion).
8. The cement nail inserted into the pre-reamed canal in the standard fashion and X-rays are confirmed to ensure ACCIN in the correct position within the canal.
  - There have been no cases of cement delamination.

### Clinical cases

#### Case 1. DH

DH: a 29-year-old male smoker who had a grade 3 open fracture of the right tibial and femoral shafts after a motor bike accident. He was managed with intramedullary nailing and staged skin grafting but developed a draining sinus post-operatively. He had been trialled with prolonged antibiotic treatment for 2 years, but the sinus failed to close, and he developed osteomyelitis before presenting to our institution. He had previously grown pseudomonas and methicillin-resistant staphylococcus aureus (MRSA).

**Fig. 3:**
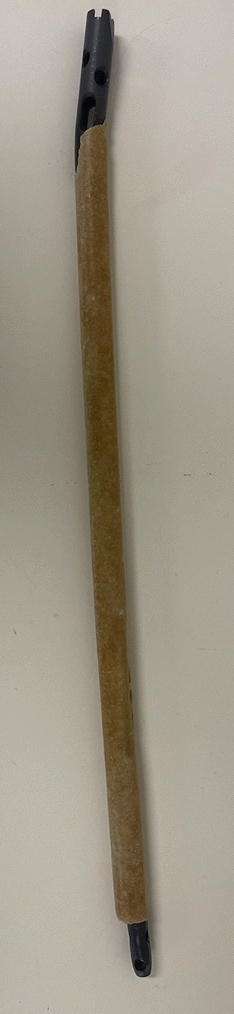
DH removed ACCIN

After a pre-operative CTA, an infectious disease and plastic surgery consult, he underwent removal of all infected hardware, sequestrectomy and removal of dead bone. An ACCIN was inserted and with a planned Masquelet antibiotic impregnated cement block and a gracilis free flap. He was treated with 9 months of oral cephalexin and ciprofloxacin and went onto union. At 3 years, his ACCIN was removed at the patient’s request without delamination of the cement from the ACCIN (see Fig. 3 DH removed ACCIN).

#### Case 2. WH

WH: a 40-year-old male smoker and intravenous drug user. He presented with a grade 3 open tibial fracture due to an earth grinder accident which was managed with staged debridement and open reduction and internal fixation with primary closure. 6 months later with chronic osteomyelitis, a large sequestrum, a discharging sinus and union of 2 cortices. He presented to his local peripheral hospital with a pre-tibial abscess and was transferred to our institution.

He was treated with initial debridement and removal of metalwork and considering the large bony defect, managed with a cast. After a CTA, an infectious diseases and plastic surgery consult, he was managed one week later with insertion of an ACCIN and gastrocnemius flap. He discharged against advice six days later and did not attend any follow-up appointments.

#### Case 3. IW

**Fig. 4:**
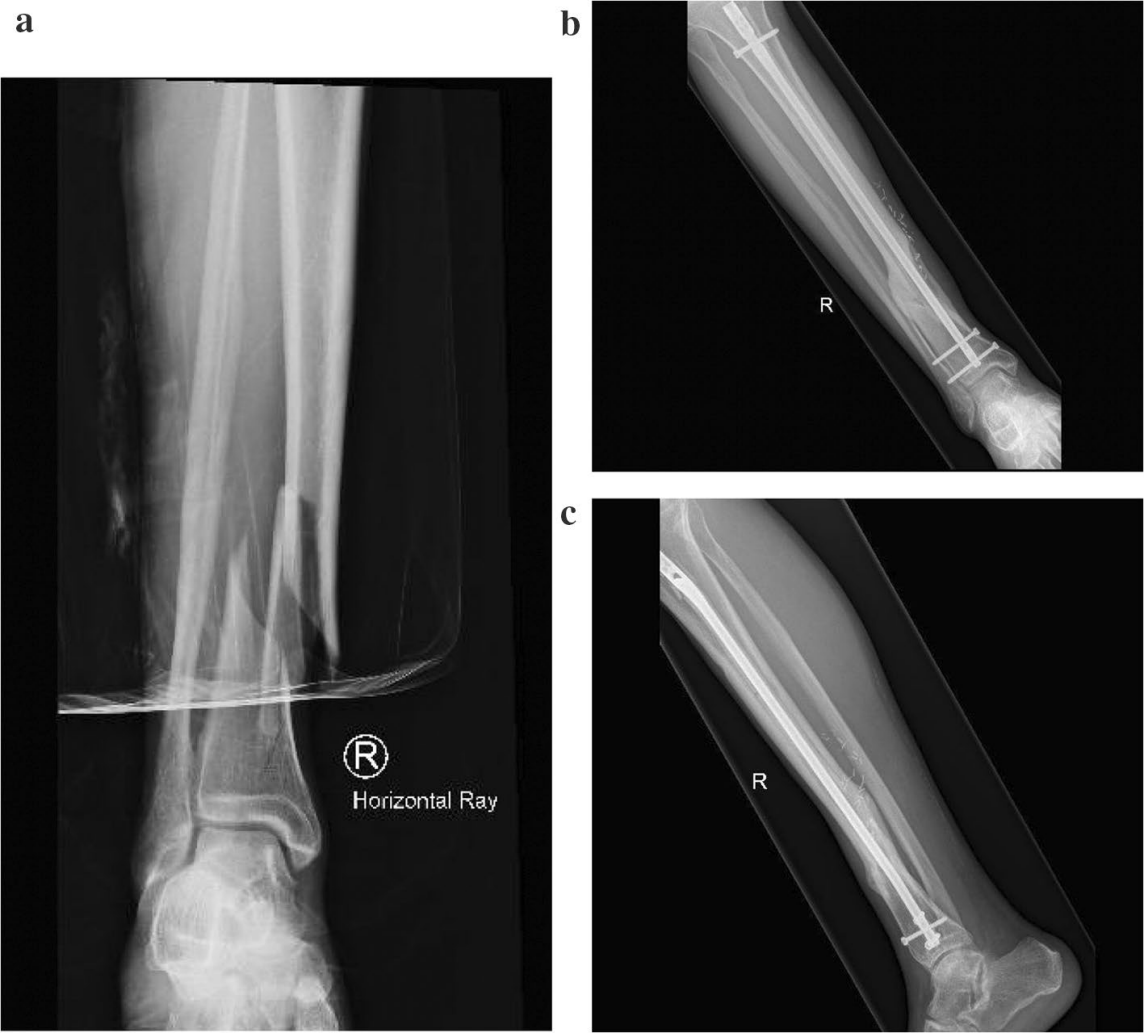
**a** IW Initial fracture. **b**–**c** IW United fracture with ACCIN

IW: a 61-year-old male heavy smoker who presented with a grade 2 open distal 1/3 tibial fracture after a fall from a bicycle (Fig. 4a IW Initial fracture). Due to his poor soft tissue quality and smoking, he was managed with a circular frame. Unfortunately, after 5 months, his sinus continued to intermittently drain, he continued to smoke, and his fracture had not united. Swabs of the sinus grew methicillin-sensitive staphylococcus aureus (MSSA) and Escherichia coli. After a pre-operative CTA, infectious disease and plastic surgery consult, he was treated with removal of his exfix, excision of his sinus, an ACCIN and an anterolateral thigh free flap. He continued oral antibiotics for 9 months, and his fracture united after 12 months (Fig. 4b–c IW United fracture with ACCIN).

## Discussion

In our small cohort, infection has been eradicated and bony union has been achieved to the best of our knowledge. A 70–100% bony union rate with eradication of infection has been reported in over 41 clinical studies [2]. ACCIN has also been shown as successful in the treatment of septic non-unions in long bones in achieving earlier weight bearing and faster rehabilitation, decreasing the number of subsequent procedures [3].

The literature has reported several different techniques of nail coating for ACCIN. Descriptions include rolling the cement coating onto the implant [10, 11], applying the cement manually using a syringe [12, 13], customised moulding made of silicon, metal and rubber [2, 14]. Pre-existing tubing was most common technique described in the literature, particularly chest tube trains of varying sizes [2]. A complication specific to ACCIN is delamination of the cement, both at the time of insertion and removal [2, 15]. Techniques to decrease the risk of this complication are to create a smooth cement mantle and over-ream the canal by 2 mm [9, 16].

A recent systematic review reported 33 different implants utilised as the core for the cement nails [2]. Early documentation of the using intramedullary guidewires, Ilizarov wires and K-wires did not favour weight bearing and bone healing [4, 16–22]. Currently, locked intramedullary nails provide the most stability for weight bearing post-operatively [2]. Even with large bone defects, ACCIN has been reported to allow simultaneous weight bearing, infection eradication and bony union (often with secondary procedures) in infected non-unions of the tibial shaft [23].

ACCIN has been used now for over two decades with success. The technique described is a practical step-by-step approach of our technique using the Ambu flexible bronchoscopy tubing. Several centres have approached us for details of the technique and the equipment used, and therefore, we have reported the details here. The current ACCIN technique described meets guidelines of the current literature of an antibiotic cement coated intramedullary locked nail to allow immediate weight bearing and local antibiotic delivery. It provides a smooth cement mantle with a precise diameter of 12 mm, allowing for 2 mm of over reaming in most tibias, to prevent delamination.

## Conclusion

We present a simple, cost-effective technique using Ambu flexible bronchoscopy tubing for creating ACCIN, to allow immediate weight bearing with local antibiotic delivery. Bronchoscopy tubing is an ideal material to create ACCIN for treatment of infected tibial non-unions.
